# Inhibition of In Vitro *Clostridioides difficile* Biofilm Formation by the Probiotic Yeast *Saccharomyces boulardii* CNCM I-745 through Modification of the Extracellular Matrix Composition

**DOI:** 10.3390/microorganisms10061082

**Published:** 2022-05-24

**Authors:** Pierre-Alexandre Lacotte, Alexis Simons, Sylvie Bouttier, Jeanne Malet-Villemagne, Valérie Nicolas, Claire Janoir

**Affiliations:** 1 INRAE, Université Paris-Saclay, AgroParisTech, Micalis Institute, 92290 Châtenay-Malabry, France; pierre-alexandre.lacotte@universite-paris-saclay.fr (P.-A.L.); alexis.simons@iutsf.org (A.S.); sylvie.bouttier@universite-paris-saclay.fr (S.B.); jeanne.malet@universite-paris-saclay.fr (J.M.-V.); 2 Laboratoire Eau, Environnement et Systèmes Urbains (Leesu), Université Paris-Est Créteil, École des Ponts ParisTech, 94010 Créteil, France; 3 Ingénierie et Plateformes au Service de l’Innovation (IPSIT), UMS IPSIT Université Paris-Saclay-US 31 INSERM-UAR 3679 CNRS, Plateforme d’Imagerie Cellulaire MIPSIT, 92290 Châtenay-Malabry, France; valerie.nicolas@universite-paris-saclay.fr

**Keywords:** *Clostridioides difficile*, biofilm, *Saccharomyces boulardii* CNCM I-745, matrix composition, eDNA

## Abstract

*Clostridioides difficile* is responsible for post-antibiotic diarrhea and most of the pseudomembranous colitis cases. Multiple recurrences, one of the major challenges faced in *C. difficile* infection (CDI) management, can be considered as chronic infections, and the role of biofilm formation in CDI recurrences is now widely considered. Therefore, we explored if the probiotic yeast *Saccharomyces boulardii* CNCM I-745 could impact the in vitro formation of *C. difficile* biofilm. Biomass staining and viable bacterial cell quantification showed that live *S. boulardii* exerts an antagonistic effect on the biofilm formation for the three *C. difficile* strains tested. Confocal laser scanning microscopy observation revealed a weakening and an average thickness reduction of the biofilm structure when *C. difficile* is co-incubated with *S. boulardii*, compared to the single-species bacterial biofilm structure. These effects, that were not detected with another genetically close yeast, *S. cerevisiae*, seemed to require direct contact between the probiotic yeast and the bacterium. Quantification of the extrapolymeric matrix components, as well as results obtained after DNase treatment, revealed a significant decrease of eDNA, an essential structural component of the *C. difficile* biofilm matrix, in the dual-species biofilm. This modification could explain the reduced cohesion and robustness of *C. difficile* biofilms formed in the presence of *S. boulardii* CNCM I-745 and be involved in *S. boulardii* clinical preventive effect against CDI recurrences.

## 1. Introduction

*Clotridioides (Clostridium) difficile* is a Gram-positive, strictly anaerobic, spore-forming rod-shaped bacterium. In Western countries, *C. difficile* is the leading cause of healthcare-associated intestinal infections in adults, although the number of community *C. difficile* infections (CDI) has been steadily increasing recent years [1]. The clinical signs range from self-resolutive mild to severe diarrhea and in extreme cases life-threatening pseudomembranous colitis [2]. Furthermore, rare complications such as fulminant colitis may require urgent medico-surgical management. A major challenge encountered with CDI is recurrences, experienced by around 25% of patients after a first episode of CDI. Moreover, a patient suffering a first recurrence has a higher risk of subsequent recurrence (e.g., 45% after a second episode) and may enter a cycle of multiple recurrences. The majority of recurrences appear to be relapses related to the persistence in the colonic niche of the strain responsible for the initial episode, although re-infections have also been identified [3,4]. First episodes of CDI are currently treated by specific antibiotherapy, fidaxomicin or vancomycin, depending on the severity of the infection and the risk factors of the patient for developing severe CDI. Metronidazole, which has been the first-choice treatment for non-severe CDI for a long time, has been recently downgraded to an alternative treatment if the two aforementioned antibiotics are unavailable [5]. In contrast, multiple relapses are often refractory to antibiotic-based treatments and are now increasingly managed by fecal microbiota transplantation. However, this approach has several drawbacks such as its significant cost, the associated infectious risks and its potential long-term side effects [6].

Besides CDI, 4% to 15% of healthy adults are asymptomatically colonized by *C. difficile* strains, either toxigenic (i.e., pathogenic strains) or non-toxigenic strains [7]. It is now recognized that asymptomatic carriers contribute to the shedding of *C. difficile* in the environment, potentially leading to cross-contamination, particularly in hospital settings. The major risk for developing CDI after *C. difficile* contamination is intestinal dysbiosis leading to disruption of the barrier effect against this pathogen. Dysbiosis is mainly induced by broad-spectrum antibiotic therapy and old age [8]. Indeed, dysbiosis promotes the germination of spores already present in the intestine (healthy carrier) or in transit (exogenous contamination). The vegetative forms then multiply, colonize the intestinal mucosa and produce the two major toxins, TcdA and TcdB. These glycosylating enzymes target Rho, Rac and Cdc42 GTPases, leading to an alteration of actin cytoskeleton and stimulating the release of pro-inflammatory cytokines. This results in severe intestinal damage and therefore clinical manifestations. In several cases, despite a specific antibiotic therapy, *C. difficile* persists in its colonic niche, hence the risk of a relapse. It was long believed that the persistence of *C. difficile* in the gut takes place as resistant spores. In recent years, however, the potential role of *C. difficile* biofilm has been discussed [9,10]. Indeed, multiple CDI relapses can be viewed as a chronic infection, and most chronic human infections are linked to the persistence of bacteria in their niche in a sessile state [11]. In vitro, *C. difficile* can form biofilms [12,13] whose extrapolymeric matrix comprises proteins, polysaccharides and eDNA [13,14,15]. Several arguments, in particular the presence of biofilm-like structures in in vitro models mimicking the digestive tract and in animal models [16,17,18,19], suggest that *C. difficile* does indeed form a biofilm in vivo. Bacteria included in biofilm structures are known to display a low susceptibility to both immune response and antibiotics, and the removal of biofilms is often very difficult [20]. Therefore, strategies contributing to hindering *C. difficile* in vivo biofilm formation or disrupting pre-formed biofilm may be a promising alternative to fight against CDI and/or recurrent CDI, and several probiotics have been shown to display properties that may impede or inhibit biofilm growth by several pathogenic bacteria [21].

*Saccharomyces boulardii* CNCM I-745 is a non-pathogenic yeast with probiotic properties [22]. Several clinical studies and meta-analyses have shown this probiotic to effectively prevent antibiotic-associated diarrhea [23,24]. Results on the prophylaxis of CDI [25,26] are more conflicting; however, a large meta-analysis supports a moderate quality evidence protective effect for *S. boulardii* in prophylaxis of first episode CDI [27]. The prophylaxis of recurrent CDI by *S. boulardii* has also been the subject of a few studies, but most have low statistical power, and the results are heterogeneous [22]. Nevertheless, a study has shown that *S. boulardii* can be used in combination with vancomycin in the treatment of recurrent CDI [26], and a recent systematic review identified a benefit of *S. boulardii* in the prevention of recurrent CDI [28]. Reasons for the beneficial effect of *S. boulardii* on the occurrence of CDI are multiple. First, *S. boulardii* treatment can reduce part of the antibiotic-induced microbiota changes [29,30] or attenuate the overreacting inflammatory immune response, thus reducing the intestinal lesions [31,32]. In addition, different specific mechanisms have been shown to explain the antagonistic effect against *C. difficile*: hydrolysis of toxins A and B by a serine protease produced by *S. boulardii* [33], stimulation of intestinal IgA raised against *C. difficile* toxin A [34], modulation of the inflammatory response to *C. difficile* toxins [35] and more recently, modulation of the bile salt profile in favor of secondary bile acids capable of inhibiting vegetative forms of *C. difficile* [36]. Another hypothesis is that the probiotic yeast *S. boulardii* CNCM I-745 could impact the formation of the *C. difficile* biofilm, potentially involved in the colonization process or the persistence of this bacterium in the gastrointestinal tract of patients [9,10] and consequently in the pathophysiology of CDI. This study explores this hypothesis and reveals that *S. boulardii* indeed has a preventive effect on the production of biofilm on inert support by *C. difficile*, presumably via a modification of the matrix composition.

## 2. Materials and Methods

### 2.1. Microorganism Strains and Growth Conditions

Three strains of *Clostridioides difficile* (CD) were used in this study: two clinical strains, i.e., strain R20291 (PCR-ribotype 027) and strain 1064 (PCR-ribotype 014/020), and the mutant strain 630Δ*erm cwp84*::*erm* (PCR-ribotype 012), an insertion mutant of the 630Δ*erm* strain for the gene *cwp84* encoding the cysteine protease Cwp84, known to produce a very robust biofilm [13]. All *C. difficile* strains were from a −80 °C frozen stock and were routinely cultured on Brain Heart Infusion (BHI, Difco) agar plates, supplemented with 3% horse blood, in anaerobic conditions at 37 °C. 

Two yeast strains were used: the probiotic yeast *Saccharomyces boulardii* (SB) CNCM I-745 was provided by Biocodex (Gentilly, France) in lyophilized powder*. Saccharomyces cerevisiae* (Sc) ATCC 9763 was used for non-probiotic yeast control. Both were grown on Sabouraud agar plates and routinely in BHI broth.

### 2.2. Preparation of Secreted Components from S. boulardii Supernatant

Secreted components from *S. boulardii* culture supernatant were prepared as previously described [31]. Briefly, 0.1 g of *S. boulardii* lyophilized powder (≈4 × 10^8^ CFU/mL) was resuspended in 10 mL RPMI and incubated for 24 h at 37 °C in aerobic conditions. The culture was then centrifugated 15 min at 7500× *g*, at 4 °C. The supernatant was collected and filtered using 0.2 µm pore size filters. 

### 2.3. Biofilm Formation

Overnight cultures in BHISG broth (BHI supplemented with 2 g/L of D-Glucose, 1 g/L of L-cystein and 5 g/L of yeast extract) of *C. difficile* strains were diluted to an approximative concentration of 1 × 10^5^ CFU/mL, and these suspensions were used as inocula for biofilm formation, for both single- (bacterium or yeast) and dual-species (bacterium and yeast) biofilms. 

*S. boulardii* lyophilized powder was resuspended in BHISG at 1% (m/V) and diluted further to obtain CD:SB ratios of 1:1, 1:10 and 1:100 (respectively, 10^5^, 10*^6^* and 10^7^ CFU/mL). *S. cerevisiae* ATCC 9763 was grown overnight in BHISG broth and then diluted to obtain the desired CD:Sc ratios. For biomass quantification and viable bacterial cells counts, single- and dual-species biofilms were grown altogether in 24-well polystyrene plates (Costar ^®^); 0.5 mL of *C. difficile* suspension at 10^5^ CFU/mL was deposited in each well, supplemented or not with 0.5 mL of *S. boulardii* suspension at 10^5^ CFU/mL (CD:SB 1:1), 10^6^ CFU/mL (CD:SB 1:10) or 10^7^ CFU/mL (CD:SB 1:100). Plates were prepared under aseptic conditions and incubated for 24 h, 48 h or 72 h in anaerobic conditions at 37 °C. Then, the culture medium was carefully removed from all wells, and the biofilms were gently washed twice with 1 mL of PBS to remove non-adherent bacteria before quantifying the biomass and the viable and culturable cells (VCC, see Section 2.4). This protocol was used for every *C. difficile* strains.

For confocal laser scanning microscopy analysis, single-species R20291 and dual-species biofilms were formed as described above except for the 24-well plates used (Greiner Bio-One Life Science, Frickenhausen, Germany).

### 2.4. Biofilm Quantification

Biofilm biomass was quantified by the classic crystal violet (CV; Thermo Fisher Scientific, Geel, Belgium) staining method, as previously described [13,19]. After the washing, biofilms were air-dried for 10 min at 37 °C before staining with 0.2% CV (*w*/*v*) for 30 min. After CV removal, wells were washed twice again with 1 mL of PBS. Biofilm biomass was solubilized with 1 mL of 80:20 ethanol/acetone (*v*/*v*) solution, scraped from the plate and quantified by the measure of OD_570_ (V-1200 Spectrophotometer, VWR).

For VCC counts, biofilms were scraped after the washing steps and resuspended in 1 mL of PBS before plating 100 µL of the homogenized and diluted suspensions on adequate media to determine the number of microorganisms. *C. difficile* CFU counts were determined by plating dilutions on BHI for single-species biofilms or on BHI supplemented with 0.03 µg/mL of amphotericin B to remove yeast presence for dual-species biofilms. *S. boulardii* and *S. cerevisiae* CFU counts were determined by plating on Sabouraud medium.

### 2.5. Confocal Laser Scanning Microscopy

The structure of the biofilm was analyzed with an inverted confocal microscope system LSM 510 META (Carl Zeiss Inc., Oberkochen, Germany) equipped with an Argon (488 nm) and Helium–Neon (543 nm) lasers and a Plan-Apochromat 20X/0.75 dry objective lens. The green and the red fluorescence emissions were collected with a 505–550 nm band-pass and a 552–670 nm band-pass emission filter, respectively, under a sequential mode. The pinhole was set at 1.0 Airy unit; 12-bit numerical images were acquired with LSM 510 software version 3.2 (Carl Zeiss Inc., Oberkochen, Germany). Observations were carried out for single-species biofilms of SB and CD (strain R20291) and for dual-species biofilms, at CD:SB ratios of 1:10 and 1:100, after 24 h and 48 h of incubation. The inoculum of CD was the same for each condition. Regarding SB, the inoculum used for single-species biofilm was the same as the inoculum in the dual-species biofilm CD + SB at 1:10 ratio. The biofilm was double stained with SYTO 9 and Propidium iodide from the LIVE/DEAD™ BacLight™ Kit (Thermo Fisher Scientific, Eugene, OR, USA). Briefly, 200 µL of both diluted fluorescent dyes (1:1000) were added per well, and the plates were incubated 15 min at room temperature prior to observation. During the Z-stack acquisition, an average of four areas for each well was analyzed. The thickness of biofilm was measured from the Z-stack acquisition. In addition, three-dimensional projections were reconstructed from x to z stacks using the software Imaris (Bitplane, Belfast, UK) 9.7.2 version.

### 2.6. Analysis of Extracellular Polymeric Substances (EPS) of the Biofilms

#### 2.6.1. Biofilm Formation

These assays have been carried out with the R20291 strain. The biofilms were prepared in 6-well plates (Costar^®^) to obtain a larger amount of biomass. Suspensions and plates were prepared as described above (see Section 2.3), except 6 mL of suspension was deposited in each well for both single- and dual-species biofilms, for which only the CD:SB ratio 1:100 has been studied. Plates were incubated at 37 °C in anaerobic conditions during 48 h.

#### 2.6.2. EPS Extraction and Quantification

Biofilms were washed twice with 1 mL of PBS to eliminate non-adherent microorganisms, and plates were dried at 37 °C for 10 min. Adherent biofilms in each well were collected and pooled into a final volume of 1.9 mL of PBS by scratching the bottom of the wells. The thick suspensions obtained were homogenized by vortexing for 1 min before being centrifuged 15 min at 5000× *g* and 4 °C. 0.2 µm filtered supernatants were used to quantify proteins, polysaccharides and extracellular DNA (eDNA). Pellets were dried overnight by lyophilisation (freeze dryer TELSTAR™ Cryodos, Terrassa, Spain) and weighted. The weight of each EPS was normalized to the dry weight of the relative biofilm used for extraction.

Protein quantification was performed using Bradford assay (Biorad, Hercules, CA, USA), according to the manufacturer instructions, with Bovine Serum Albumin (BSA, Euromedex, Souffelweyersheim, France) used as standard.

Quantification of polysaccharides was performed by phenol–sulfuric acid assay. Briefly, samples were processed as follows: (i) 100 µL of EPS extract solution was mixed with 100 µL of deionised water, 200 µL of phenol 5% (Sigma, Saint Louis, MI, USA) and 1 mL of sulfuric acid (Sigma, Saint Louis, MI, USA); (ii) the mix was incubated 5 min at 100 °C and then 30 min at room temperature in the dark; (iii) dosage was carried out by absorbance measurements at 490 nm, using glucose solution (AnalaR NORMAPUR^®^) as standard.

The eDNA was first purified from filtrate samples using phenol/chloroform/isoamyl alcohol (25:24:1, *v*/*v*, Invitrogen™, Carlsbad, CA, USA), then precipitated by absolute ethanol (VWR) overnight at −20 °C. After centrifugation (16,000× *g*, 4 °C, 30 min), the DNA pellet was washed with 150 µL of 70% ethanol and centrifuged at 16,000× *g* 4 °C for 2 min. The pellet obtained was dried at room temperature for 10 min and dissolved into 20 µL of deionised water. DNA concentration was measured by Nanodrop™ (Thermo Scientific™, Wilmington, DE, USA) at λ = 260 nm.

#### 2.6.3. Biofilm Dispersion Using DNase I Treatment

These assays were performed using biofilms formed in 24-well plates after an incubation at 37 °C for 48 h (see Section 2.3). After 2 washes with 1 mL of PBS each, plates were dried at 37 °C for 10 min. One milliliter of DNase (Sigma, Saint Louis, MI, USA) at 25 µg/mL or DNase buffer alone (TrisHCl 10 mM; MgCl_2_ 2.5 mM; CaCl_2_ 0.1 mM) was added into the wells, before 1 h of incubation at 37 °C under anaerobic conditions. Wells were washed again twice (2 × 1 mL of PBS) to remove the fraction of the biofilm that has been dispersed by the enzymatic treatment. The remaining biomass was quantified by crystal violet staining (see Section 2.4).

### 2.7. Statistical Analysis

All statistical analyzes were carried out using the Mann–Whitney non-parametric test.

## 3. Results

### 3.1. Effect of S. boulardii CNCM I-745 on C. difficile R20291 Biofilm

**Figure 1:**
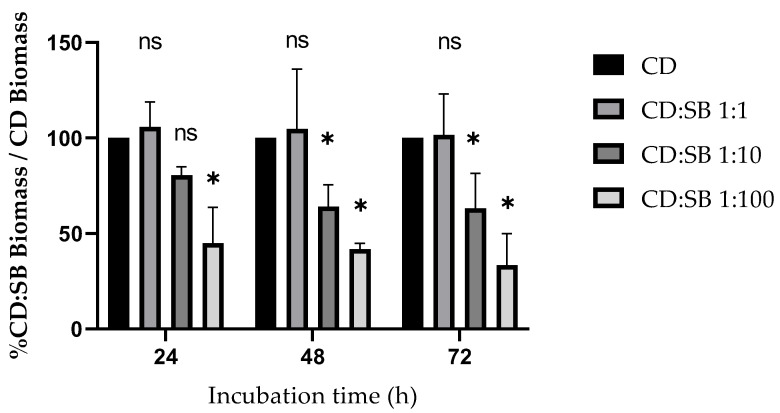
Biofilm formation inhibition: relative reduction of dual-species biofilm biomass compared to the biomass of the single-species (CD, strain R20291) biofilm, at different CD:SB ratios and incubation durations. Means were calculated from at least four different biological replicates. Asterisks indicate statistical differences with single-species (CD) biofilm biomass determined by a Mann–Whitney test (* *p* ≤ 0.05; ns, not significant, *p* > 0.05).

To evaluate the impact of live *S. boulardii* on *C. difficile* biofilm production, both were co-cultured at CD:SB ratios 1:1, 1:10 and 1:100 in 24-well plates for 24 h, 48 h and 72 h. The biomasses of the resultant biofilms were quantified using the crystal-violet staining method. For each assay, mean absorbance was calculated from two separate measurements. The proportion of biofilm reduction was estimated by the ratio of the biomass of the dual-species biofilm over the biomass of the single-species biofilm of *C. difficile.* No matter the incubation duration, *S. boulardii* had no effect on *C. difficile* biofilm biomass for the 1:1 CD:SB ratio. For the other conditions tested (defined by the ratio CD:SB and the duration of biofilm incubation), the effect of *S. boulardii* on *C. difficile* biofilm constantly resulted in a decrease of the dual-species biofilm biomass compared to the single-species CD biofilm (Figure 1). Moreover, the decrease was both ratio- and incubation-time dependent. For ratios 1:10 and 1:100, *S. boulardii* co-cultured with *C. difficile* significantly decreased biofilm biomass compared to the single-species biofilm of *C. difficile* (Mann–Whitney test, *p* < 0.01), except for the 24 h biofilm for ratio 1:10 (Mann–Whitney test, *p* > 0.01). This reproducible antagonist effect is represented in Figure 1 as the mean relative biomass of dual-species biofilms in each condition. The quantification of the single-species SB biofilm showed that the probiotic yeast formed a low biofilm at each time point tested (Figure S1).

**Figure 2:**
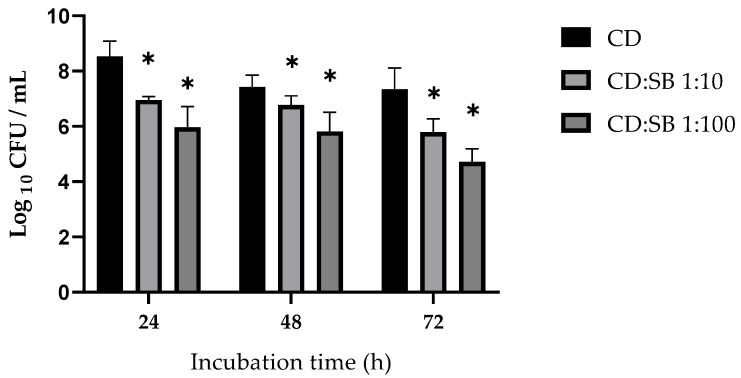
Biofilm formation inhibition: reduction of *C. difficile* VCC in dual-species biofilms compared to single-species (CD) biofilms at different ratios and incubation durations. Washed biofilms were scraped and resuspended in 1 mL of PBS before plating 100 µL of dilutions on BHI for single-species CD biofilms or on BHI supplemented with 0.03 µg/mL of amphotericin B for dual-species (CD + SB) biofilms to determine the number of CFU. Means were calculated from at least four different biological replicates. Asterisks indicate statistical differences of *C. difficile* viability with the single-species (CD) biofilm determined by a Mann–Whitney test (* *p* ≤ 0.05).

To investigate the impact of *S. boulardii* on *C. difficile* viability, CFU counts were carried out on single- and dual-species biofilms. As previously observed for biomass, *S. boulardii* had no effect on bacterial cell viability for the CD:SB 1:1 ratio, regardless of the incubation time. For CD:SB ratios of 1:10 and 1:100, the number of viable *C. difficile* cells was significantly reduced by co-incubation with *S. boulardii,* from 24 h to 72 h (Figure 2).

Since *S. boulardii* secreted compounds have been reported to hinder biofilm formation by *Candida albicans* [37], we investigated the impact of supernatant from *S. boulardii* on *C. difficile* biofilm. A total of 500 µL of the preparation containing secreted components was added to 500 µL of *C. difficile* grown overnight in BHI 2X and incubated in 24-well plates for 48 h, before biomass quantification by crystal violet staining and bacterial CFU count. The trend toward reduced biomass observed after incubation of *C. difficile* with *S. boulardii* secreted components was not significant (Mann–Whitney test, *p* > 0.01), and no reduction in bacterial viability was noticed. Therefore, the supernatant from *S. boulardii* cultures seems to have no effect on *C. difficile* biofilm production (Figure S2).

### 3.2. Impact of S. boulardii CNCM I-745 on the Biofilm Production by Other Strains of C. difficile

To confirm our observations on *C. difficile* strain R20291, we conducted the same experiments on two other strains of *C. difficile* belonging to prevalent PCR-ribotypes, the strain 1064 and the mutant strain 630Δ*erm cwp84*::*erm* (PCR-ribotype 14/20 and 012, respectively).

**Figure 3:**
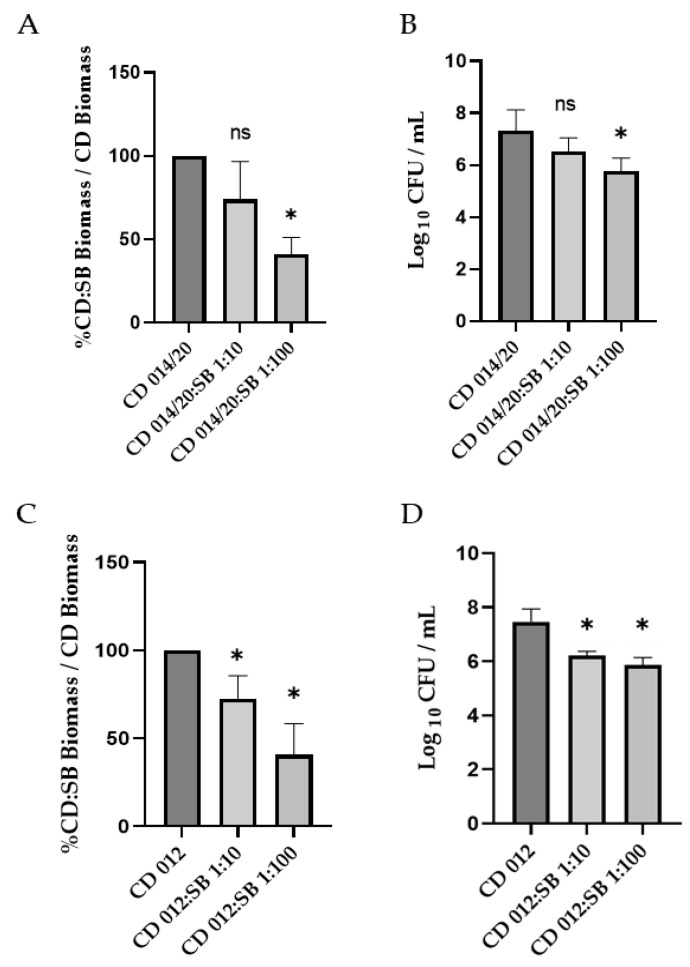
Impact of *S. boulardii* on strains 1064 (PCR ribotype 014/020) and 630Δerm *cwp84*::*erm* (PCR ribotype 012) biofilms: (**A**,**C**) biomass quantification assays; and (**B**,**D**) CFU counts were performed on dual-species biofilms and compared to single-species biofilms. Means were calculated from at least four different replicates. * *p* < 0.05, Mann–Whitney test; ns, not significant, *p* > 0.05.

The optimal incubation duration for single-species biofilm production was 48 h for strain 1064 and 72 h for the mutant 630Δ*erm cwp84*::*erm*. Dual-species biofilm formation was studied with CD:SB ratios 1:10 and 1:100. For strain 1064 incubated with *S. boulardii* at a ratio CD:SB 1:100, dual-species biofilm had significantly decreased biomass quantities and viable bacterial counts compared to the single-species biofilm (Mann–Whitney test, *p* < 0.01). Neither biomass amount nor viable bacterial cells were significantly reduced in ratio 1:10 biofilms (Figure 3A,B).

For mutant 630Δ*erm cwp84*::*erm*, we observed a significantly decreased biomass amount together with a reduced viable *C. difficile* counts for both ratios 1:10 and 1:100 (Mann–Whitney test, *p* < 0.05) (Figure 3C,D).

### 3.3. Specificity of the Antagonistic Effect of S. boulardii CNCM I-745 toward C. difficile Biofilm

To demonstrate the specificity of *S. boulardii* CNCM I-745 impact on *C. difficile* biofilm, we investigated the effect of *S. cerevisiae* ATCC 9763 (Sc), a non-probiotic yeast with high genetic similarities to *S. boulardii*. *S. cerevisiae* was therefore co-cultured with *C. difficile* to ratio CD:Sc 1:100 for 48 h. We observed that mixed biofilms CD:Sc had enhanced biomass amount compared to mono-species CD biofilms. These observations might be related to *S. cerevisiae* inherent biofilm production properties. Indeed, single Sc biofilm displays high biomass (Figure S3), limiting the comparison with mixed biofilms. However, the count of viable *C. difficile* cells within dual-species biofilms Sc:CD was the same as in single-species biofilm (Figure S3), clearly showing that *S. cerevisiae* does not display the same effect as *S. boulardii* CNCM I-745 on the biofilm formation by *C. difficile*.

### 3.4. Visualization of Biofilms by Confocal Laser Scanning Microscopy

**Figure 4:**
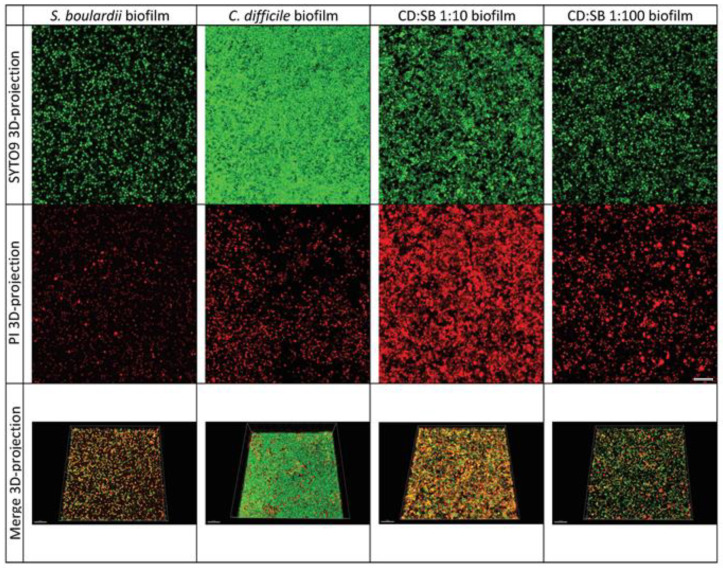
Representative images of CLSM observation of single-species *S. boulardii* biofilm, single-species *C. difficile* biofilm, dual-species CD + SB biofilms at CD:SB ratio 1:10 and 1:100, after 48 h of incubation; 3D representations of the biofilm stained by SYTO 9 and propidium iodide (first and second line, respectively) and a Merge representation of the 3D biofilm structures (third line) are shown (scale bar: 30 µm).

CLSM coupling with fluorescent live/dead staining is a powerful tool for analyzing the architecture of biofilm structure. Representative images of 24 h and 48 h biofilms are presented in Figure 4 and Figure S4, respectively. Regarding 48 h biofilms, the mono-species biofilm formed by *S. boulardii* shows a relatively sparse yeast community, with numerous holes comprising mainly live *S. boulardii*. In contrast, the single-species *C. difficile* biofilm (R20291 strain) was highly homogeneous with a densely packed community of aggregated bacterial cells and few holes; the surface coverage was high, and bacteria were mostly embedded as living cells. The image of the dual-species biofilm obtained for a CD:SB ratio 1:10 allowed us to differentiate bacteria from yeasts easily. This biofilm was also rather dense, with a coverage of almost the whole surface, despite some holes (see merge 3D-projection). The general appearance of the 1:100 CD:SB dual-species biofilm was similar to that of the *S. boulardii* mono-species biofilm, with a particularly low substratum coverage due to numerous holes. This biofilm thus appeared to be less dense than the *C. difficile* mono-species biofilm and the CD:SB 1:10 dual-species biofilm. Except for the dual-species CD:SB 1:10 biofilm, a low ratio of micro-organisms was stained by propidium iodide, suggesting most of them to be alive in the biofilm structures. A careful observation of images revealed that most of the dead (or enough damaged) microorganisms were yeasts in dual-species biofilm, for both 1:10 and 1:100 ratios. Therefore, the dual-species biofilm appeared to be less robust and homogeneous than the *C. difficile* single-species biofilm, especially for the CD:SB ratio 1:100.

Briefly, the CLSM observation of 24 h single- and dual-species biofilm structures (Figure S4) gave similar results: the density, as well as the surface coverage, of both CD:SB 1:10 and 1:100 biofilms were substantially reduced compared to *C. difficile* single-species biofilm, which already shows the structure of a mature biofilm after 24 h of incubation. The overall weakening of dual-species biofilm therefore begins quite early during the biofilm formation.

**Figure 5:**
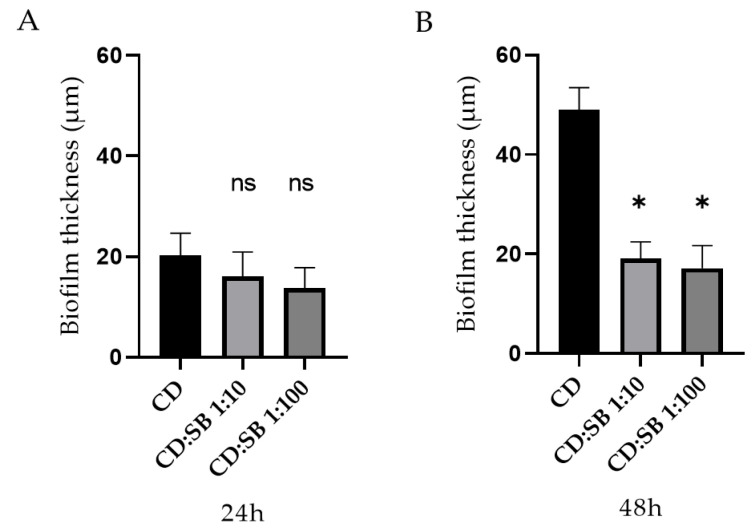
Average thickness of 24 h biofilm (**A**) and 48 h biofilm (**B**) for single-species (CD) and dual-species biofilms (CD + SB) produced in vitro (* *p* < 0.05, Mann–Whitney test; ns, not significant, *p* > 0.05).

The average thickness for 24 h and 48 h single- and dual-species biofilms were calculated from these images (Figure 5). For 24 h biofilm structures, no difference appears between single-species *C. difficile* biofilm and dual-species biofilms, whatever the CD:SB ratio tested. However, after 48 h of incubation, the value of the average thicknesses of the dual-species biofilm were significantly lower than that measured for single-species *C. difficile* biofilm (*p* < 0.05) (Figure 5).

### 3.5. Characterization of the EPS from Single- and Dual-Species Biofilms

#### 3.5.1. Quantification of the EPS from Biofilms after 48 h Growth

**Figure 6:**
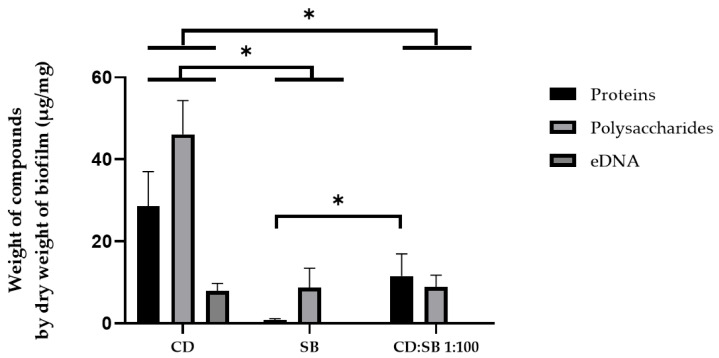
Quantification of the weight of polysaccharides, proteins and eDNA in the extracellular matrix from single- (*C. difficile*, *S. boulardii*) and dual-species (CD + SB at ratio 1:100) biofilms (after 48 h of incubation). Means and standard errors have been calculated with four independent replicates (* *p* < 0.05, Mann–Whitney test).

Respective ratios of polysaccharides, proteins and eDNA in the extracellular matrix for both single- (CD alone, SB alone) and dual-species (CD + SB at ratio 1:100) biofilms are shown in Figure S5. Briefly, *C. difficile* single-species biofilms were composed mainly of polysaccharides and proteins (representing together around 90% of the matrix composition), the residual component (≈10%) being eDNA. In comparison, yeast mono-species biofilm matrix comprises on average 90% of polysaccharides and 10% proteins, with no eDNA detected in our conditions. The main feature of the dual-species CD:SB 1:100 biofilm matrix composition was the absence of eDNA (or, at least, at an amount below the detection threshold). To compare the weight of EPS from different biofilms, EPS weight values were normalized to the dry weight of the parental biofilm (Figure 6). Extracellular matrix of single-species CD biofilms was composed of polysaccharides (46.14 ± 8.23 µg/mg of dry biomass), proteins (26.56 ± 8.45 µg/mg of dry biomass) and eDNA (7.92 ± 1.83 µg/mg of dry biomass). In contrast, the matrix of mono-species biofilm of *S. boulardii* had a different composition of matrix, composed of polysaccharides (8.73 ± 4.73 µg/mg of dry biomass), and a low quantity of proteins (0.75 ± 0.43 µg/mg of dry biomass); the eDNA quantity was below the detection threshold (inferior to 2 µg/mg of dry biomass). Concerning dual-species CD + SB biofilms at ratio 1:100, the extracellular matrix was composed almost equally of proteins (11.55 ± 5.43 µg/mg of dry biomass) and polysaccharides (8.87 ± 2.88 µg/mg of dry biomass) but eDNA was not detected. The amount of each EPS in single-species CD biofilms was significantly higher than those of both single-species (SB) and dual-species biofilms. The co-incubation of *C. difficile* with *S. boulardii* leads to a weaker biofilm, characterized by a reduced production of extracellular matrix and a lack of detectable eDNA.

#### 3.5.2. Impact of the eDNA in the Cohesiveness of Mono- and Dual-Species Biofilms

**Figure 7:**
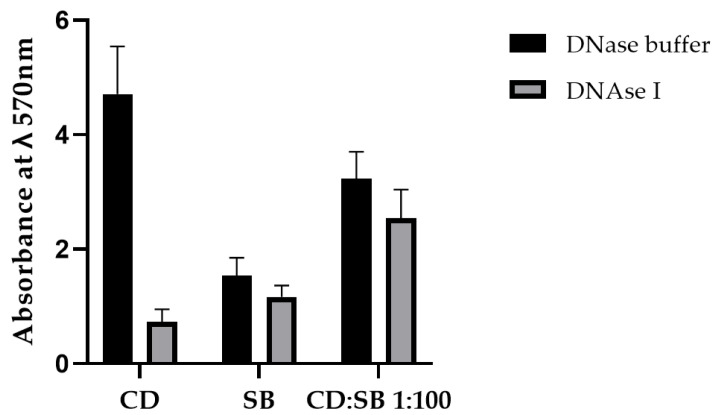
Biomass quantification by crystal violet staining of single- (*C. difficile*, *S. boulardii*) and dual-species (CD + SB at ratio 1:100) biofilms (after 48 h of incubation) after a 1 h treatment with (i) DNase buffer (control) or (ii) DNase I at 25 µg/mL final. Means and standard errors have been calculated from three independent replicates.

Enzymatic dispersion assays were carried out to evaluate the part of the eDNA to the cohesiveness of single- and dual-species biofilms after 48 h of incubation. Treatment by DNase I at 25 µg/mL induced a strong decrease in the adherent biomass for single-species CD biofilms (Figure 7). In contrast, no difference was observed between buffer and DNase treatment for the single-species yeast biofilm and the dual-species (CD:SB 1:100) biofilms, consistent with results of extracellular matrix compounds quantification that showed a detectable presence of eDNA only in single-species *C. difficile* biofilm condition. This observation confirmed that *S. boulardii* may have a specific modulatory effect on the *C. difficile* biofilm matrix composition for dual-species CD:SB biofilm.

## 4. Discussion

Recurrences are one of the major challenges faced in *C. difficile* infection management. These are mostly relapses, and multiple CDI relapses can be considered as chronic infections. As such, the role of biofilm formation in the long-term colonization of the human colon is now widely considered [9,10]. In vitro, *C. difficile* vegetative cells included within the biofilms show a decreased susceptibility to anti-*C. difficile* antibiotics such as vancomycin and fidaxomicin [38], and this could result in therapeutic failure.

The probiotic yeast *Saccharomyces boulardii* CNCM I-745 has been shown to have preventive activity against enteric pathogens, including *C. difficile*, and exerts an antagonistic effect on in vitro formation of biofilm by *Candida albicans*, a fungal commensal of the human intestinal microbiota that can be responsible for opportunistic infections [39]. We therefore studied the impact of *S. boulardii* on the formation of biofilm structures by *C. difficile* on abiotic surfaces.

To that aim, we first studied the robustness and the architecture of biofilms formed on polystyrene surfaces either as single-species (CD or SB) biofilms or as dual-species (CD + SB) biofilms, using the strain R20291, a clinically relevant 027 *C. difficile* strain, able to form a moderate-biofilm as previously described [40]. Our results showed that co-incubation of live *S. boulardii* during the formation of biofilm by *C. difficile* impacts this process negatively, leading in the dual-species biofilms, to a reduction of the biomass produced and a reduced bacterial viability as compared to *C. difficile* single-species biofilm. This antagonist effect is dose- and time-dependent since no impact was seen at the 1:1 CD:SB ratio, whatever the duration of the co-cultures, in contrast to the 1:10 and even more 1:100 ratios. With these two ratios, we observed a moderate to dramatic reduction of the dual-species biofilm biomass after 48 h and 72 h of incubation. The presence of *S. boulardii* also causes at both ratios (1:10 and 1:100) a statistically significant reduction of the viability of *C. difficile* included in the biofilm, from 24 h of incubation.

By confocal microscopy observation, we also characterized a dual-species biofilm with less aggregated microorganisms than the mono-species biofilm of *C. difficile*, together with the presence of holes and a low coverage substratum, suggesting a weakening of the biofilm structure when *C. difficile* is co-incubated with *S. boulardii*. In addition, the average thickness of this structure is significantly reduced in the 48 h dual-species biofilm, consistent with the reduction of the biomass and viable bacteria count described above. Thus, *S. boulardii* modulates the quality and the integrity of the biofilm formed by the *C. difficile* strain R20291.

The antagonist effect of *S. boulardii* on the biofilm formation by *C. difficile* was then confirmed on two other strains belonging to two different PCR-ribotypes (012 and 014/020) that have circulated in Europe in recent years and that were chosen owing to their abilities to form robust single-species biofilm [13,40]. Although we observed some subtle differences between the two strains, co-incubation with live *S. boulardii* leads to both a decrease in the global biofilm biomass and a reduced bacterial viability for these two strains. Thus, the antagonist effect displayed by *S. boulardii* on *C. difficile* biofilm formation seems to be a shared feature among different strains of *C. difficile*. Moreover, we assume that this effect is specific to the probiotic yeast *S. boulardii* CNCM I-745, at least the effect on the bacterial viability, owing to the results observed for the dual-species biofilms formed by *C. difficile* and the non-probiotic yeast strain, *S. cerevisiae* ATCC 9763. Indeed, we showed that co-incubation of *C. difficile* with *S. cerevisiae* does not change the bacterial (strain R20291) load at all in dual-species biofilm as compared to the single-cell (CD) biofilm, while in the same conditions, the presence of *S. boulardii* leads to a decrease of almost 2 log_10_ of viable *C. difficile* cells (*p* < 0.01). This observation is of importance since the antibiofilm activity of probiotics could be related to different mechanisms, for example, generation of deleterious environmental conditions (e.g., pH), competition for adhesion sites on the surface or competition for nutrients, but also secretion of metabolites with antibacterial activity (e.g., bacteriocins, organic acids) or able to modify the biofilm formation signaling pathways of pathogenic bacteria [21,41]. For example, *S. boulardii* CNCM I-745 secretes capric acid that has been shown to partially inhibit adhesion and biofilm formation of *C. albicans* on plastic surfaces and reduce candidal virulence factor production [37]. Antibiofilm microorganisms could also affect primary adhesion by modulating the physicochemical properties of pathogenic bacteria such as hydrophobicity and auto-aggregation ability, usually the result of alteration of the expression of key surface structures required for surface colonization.

Thus, in an attempt to decipher the mechanisms involved in the adverse impact of *S. boulardii* on *C. difficile* biofilm, we studied the impact of a 24 h culture supernatant of *S. boulardii* on the formation of *C. difficile* biofilm. Surprisingly, we observed a not significant low and variable inhibitory effect of the supernatant on biomass production in CD biofilm, whereas no impact was detected on cell viability. It therefore seems unlikely that the negative effect observed is due to a yeast secreted product; thus, the global inhibitory effect of *S. boulardii* on *C. difficile* biofilm formation might require direct contact between the yeast and the bacterium. We could also hypothesize that the antagonistic effect is linked to a nutritional competition between the two microorganisms which results in a growth limitation of *C. difficile* and, consequently, in a reduced production of matrix by the bacterial community. The reduction of bacterial viability was also observed in planktonic co-cocultures. Comparison of *C. difficile* VCC between single CD culture and (CD + SB) co-cultures in BHISG showed that the presence of live yeasts accelerates the reduction of bacterial CFU counts as soon as 24 h of incubation. Nevertheless, this result may be misinterpreted due to an unexpected effect as seen by optic microscopic observation: SB binds to CD and induces a bacterial morphology defect with an important bacterial elongation, which could be related to a negative impact of SB on the septum division (Figure S6). Thus, the bacterial CFU counts may be in part underestimated. Of note, by CLSM, despite a close examination of our images, we never observed the same bacilli elongation in biofilm. Moreover, two observations suggest that the negative effect of SB on CD biofilm is not only related to nutritional competition. First, as previously mentioned, no impact on *C. difficile* viability was noticed when co-incubated with *S. cerevisiae*, which has a close metabolic behavior to that of *S. boulardii*. Second, in the dual-species biofilms, we observed a low growth of *S. boulardii* within the dual-species biofilm, likely insufficient to cause a nutrient deficiency in the medium.

Additional analyses were performed on 6 h single- or dual-species biofilms to explore the hypothesis of inhibition of *C. difficile* adhesion to the surface in the presence of *S. boulardii*. The crystal violet staining was not sensitive enough to be used, but we quantified the adherent bacteria. As no difference was observed between the single- and the dual-species biofilm (6.04 × 10^6^ and 6.58 × 10^6^ adherent live bacterial cells, respectively), we could not relate the antagonistic effect of *S. boulardii* on *C. difficile* biofilm formation to a decrease of the initial bacterial adhesion but rather to a modulation in the later stages of biofilm formation.

Finally, considering that microorganism interferences can modify the matrix composition, we quantified the matrix components in mono-species (CD and SB) biofilms and dual-species biofilm formed at the 1:100 CD:SB ratio after 48 h of incubation. Polysaccharides, proteins and eDNA are the main matrix components described for *C. difficile* biofilms [10]. Not surprisingly, the quantities of the three components were significantly reduced in the dual-species biofilm compared to the single-species biofilm of *C. difficile* relevant to the decreased bacterial VCC in the former. More interestingly, we highlighted modifications in the relative abundance of the three matrix components, an increase for proteins and a decrease for polysaccharides and eDNA in the dual-species biofilm. The apparent absence of eDNA is of particular interest because this compound has an important role in the cohesion of the biofilms of many pathogenic bacteria [42,43,44,45]. Little work has been carried out on the role of eDNA in the *C. difficile* biofilm, but two recent publications strongly suggest a key role in the cohesiveness of *C. difficile* biofilm too [14,46]. As the overall decrease of extrapolymeric matrix may lead to an eDNA amount below the method detection threshold and a further biased interpretation of the results, we performed a DNase dispersion test. Treatment with DNase I decreased single-species CD biofilm by 84% ± 4% and dual-species (CD + SB) biofilm by 21% ± 4%, thus confirming the decrease of eDNA relative abundance in the dual-species biofilm. This could explain the fragility of these structures revealed by confocal microscopy. Mechanisms of eDNA release during *C. difficile* biofilm formation are not well defined but could involve phage-mediated release or autolysis [10]. The mean by which *S. boulardii* can modulate eDNA amount in the matrix of *C. difficile* biofilm thus remains to be determined but could be related to degradation of eDNA or a local inhibition of the processes allowing its release into the external environment. This last hypothesis is more likely since preliminary experiments showed, on the one hand that live *S. boulardii* are not able to disperse a 24 h or 48 h pre-formed *C. difficile* biofilm, and on the other hand that no extracellular DNase is released by this yeast, suggesting inhibition of eDNA release more than a degradation of eDNA already released.

To our knowledge, this study is the first that specifically examines the antagonistic effect of a recognized probiotic on the biofilm formation process of *C. difficile*. Our results showed unambiguously that *S. boulardii* CNCM I-745 exerts an antagonistic effect on the formation of the *C. difficile* biofilm, in terms of biomass produced, bacterial viability and architecture of the biofilm structure. Two mechanisms may explain this effect. Although presumably not restricted to a sessile state, *S. boulardii* hinders the growth of *C. difficile*, likely by nutritional competition, leading to an overall decrease of extrapolymeric matrix production. In addition, *S. boulardii* hampers the secretion of eDNA, a key component for the structural integrity of the biofilm. Similar mechanisms have been proposed recently to explain the antagonistic effect of *Lactobacillus casei* on *C. albicans* biofilm formation [47]. In addition, eDNA (as well as exopolysaccharides) of the biofilm matrix can act as a barrier to diffusion and thus reduce the penetration of antibiotics into biofilms [21]. Thus, a reduced eDNA amount in *C. difficile* biofilm matrix may also facilitate the bactericidal activity of an antibiotic against this important intestinal pathogen. Further studies focusing on antibiotics associated with *S. boulardii* treatment efficacy on *C. difficile* biofilms could provide new insights into the management of patients with recurrent CDI.

## Supplementary Materials

The following supporting information can be downloaded at: https://www.mdpi.com/article/10.3390/microorganisms10061082/s1, Figure S1: Biomass of mono-species biofilms produced by *S. boulardii* over time, as measured by crystal violet staining. Figure S2: Impact of supernatant from *S. boulardii* culture on *C. difficile* biofilm. Figure S3: Biomass production (A); and bacterial VCC count (B). Figure S4: Representative images of CLSM observation of single-species *S. boulardii* biofilm, single-species *C. difficile* biofilm, dual-species CD + SB biofilms at CD:SB ratio 1:10 and 1:100 after 24 h of incubation. Figure S5: Ratios of polysaccharides, proteins and eDNA in the composition of extracellular matrix from single- (CD alone, SB alone) and dual-species (CD:SB, ratio 1:100) biofilms. Figure S6: Optical microscopic examination after Gram staining of 24 h planktonic co-cultures at a ratio CD:SB 1:10.

## References

[1] Fu Y., Luo Y., Grinspan A.M. (2021). Epidemiology of community-acquired and recurrent *Clostridioides difficile* Infection. Ther. Adv. Gastroenterol..

[2] Martin J.S.H., Monaghan T.M., Wilcox M.H. (2016). *Clostridium difficile* infection: Epidemiology, diagnosis and understanding transmission. Nat. Rev. Gastroenterol. Hepatol..

[3] Figueroa I., Johnson S., Sambol S.P., Goldstein E.J.C., Citron D.M., Gerding D.N. (2012). Relapse versus reinfection: Recurrent *Clostridium difficile* infection following treatment with fidaxomicin or vancomycin. Clin. Infect. Dis..

[4] Cho J., Cunningham S., Pu M., Lennon R.J., Dens Higano J., Jeraldo P., Sampathkumar P., Shannon S., Kashyap P.C., Patel R. (2021). *Clostridioides difficile* whole-genome sequencing differentiates relapse with the same strain from reinfection with a new strain. Clin. Infect. Dis..

[5] Van Prehn J., Reigadas E., Vogelzang E.H., Bouza E., Hristea A., Guery B., Krutova M., Norén T., Allerberger F., Coia J.E. (2021). European Society of Clinical Microbiology and Infectious Diseases: 2021 Update on the treatment guidance document for *Clostridioides difficile* infection in adults. Clin. Microbiol. Infect..

[6] Marcella C., Cui B., Kelly C.R., Ianiro G., Cammarota G., Zhang F. (2021). Systematic Review: The global incidence of faecal microbiota transplantation-related adverse events from 2000 to 2020. Aliment. Pharmacol. Ther..

[7] Crobach M.J.T., Vernon J.J., Loo V.G., Kong L.Y., Péchiné S., Wilcox M.H., Kuijper E.J. (2018). Understanding *Clostridium difficile* colonization. Clin. Microbiol. Rev..

[8] Smits W.K., Lyras D., Lacy D.B., Wilcox M.H., Kuijper E.J. (2016). *Clostridium difficile* infection. Nat. Rev. Dis. Primer.

[9] Frost L.R., Cheng J.K.J., Unnikrishnan M. (2021). *Clostridioides difficile* biofilms: A mechanism of persistence in the gut?. PLOS Pathog..

[10] Meza-Torres J., Auria E., Dupuy B., Tremblay Y.D.N. (2021). Wolf in sheep’s clothing: *Clostridioides difficile* biofilm as a reservoir for recurrent infections. Microorganisms.

[11] Bjarnsholt T., Alhede M., Alhede M., Eickhardt-Sørensen S.R., Moser C., Kühl M., Jensen P.Ø., Høiby N. (2013). The in vivo biofilm. Trends Microbiol..

[12] Dapa T., Unnikrishnan M. (2013). Biofilm formation by *Clostridium difficile*. Gut Microbes.

[13] Pantaléon V., Soavelomandroso A.P., Bouttier S., Briandet R., Roxas B., Chu M., Collignon A., Janoir C., Vedantam G., Candela T. (2015). The *Clostridium difficile* protease Cwp84 modulates both biofilm formation and cell-Surface properties. PLoS ONE.

[14] Dawson L.F., Peltier J., Hall C.L., Harrison M.A., Derakhshan M., Shaw H.A., Fairweather N.F., Wren B.W. (2021). Extracellular DNA, cell surface proteins and c-Di-GMP promote biofilm formation in *Clostridioides difficile*. Sci. Rep..

[15] Semenyuk E.G., Laning M.L., Foley J., Johnston P.F., Knight K.L., Gerding D.N., Driks A. (2014). Spore formation and toxin production in *Clostridium difficile* biofilms. PLoS ONE.

[16] Crowther G.S., Chilton C.H., Todhunter S.L., Nicholson S., Freeman J., Baines S.D., Wilcox M.H. (2014). Development and validation of a chemostat gut model to study both planktonic and biofilm modes of growth of *Clostridium difficile* and human microbiota. PLoS ONE.

[17] Normington C., Moura I.B., Bryant J.A., Ewin D.J., Clark E.V., Kettle M.J., Harris H.C., Spittal W., Davis G., Henn M.R. (2021). Biofilms harbour *Clostridioides difficile*, serving as a reservoir for recurrent infection. Npj Biofilms Microbiomes.

[18] Semenyuk E.G., Poroyko V.A., Johnston P.F., Jones S.E., Knight K.L., Gerding D.N., Driks A. (2015). Analysis of bacterial communities during *Clostridium difficile* infection in the mouse. Infect. Immun..

[19] Soavelomandroso A.P., Gaudin F., Hoys S., Nicolas V., Vedantam G., Janoir C., Bouttier S. (2017). Biofilm structures in a mono-associated mouse model of *Clostridium difficile* infection. Front. Microbiol..

[20] Del Pozo J.L. (2018). Biofilm-related disease. Expert Rev. Anti Infect. Ther..

[21] Miquel S., Lagrafeuille R., Souweine B., Forestier C. (2016). Anti-biofilm activity as a health issue. Front. Microbiol..

[22] Kaźmierczak-Siedlecka K., Ruszkowski J., Fic M., Folwarski M., Makarewicz W. (2020). *Saccharomyces boulardii* CNCM I-745: A non-bacterial microorganism used as probiotic agent in supporting treatment of selected diseases. Curr. Microbiol..

[23] McFarland L.V. (2010). Systematic review and meta-analysis of *Saccharomyces boulardii* in adult patients. World J. Gastroenterol. WJG.

[24] Szajewska H., Kołodziej M. (2015). Systematic review with meta-analysis: *Saccharomyces boulardii* in the prevention of antibiotic-associated diarrhoea. Aliment. Pharmacol. Ther..

[25] McFarland L.V., Surawicz C.M., Greenberg R.N., Fekety R., Elmer G.W., Moyer K.A., Melcher S.A., Bowen K.E., Cox J.L., Noorani Z. (1994). A randomized placebo-controlled trial of *Saccharomyces boulardii* in combination with standard antibiotics for *Clostridium difficile* disease. JAMA.

[26] Surawicz C.M., McFarland L.V., Greenberg R.N., Rubin M., Fekety R., Mulligan M.E., Garcia R.J., Brandmarker S., Bowen K., Borjal D. (2000). The search for a better treatment for recurrent *Clostridium difficile* disease: Use of high-dose vancomycin combined with *Saccharomyces boulardii*. Clin. Infect. Dis..

[27] Goldenberg J.Z., Yap C., Lytvyn L., Lo C.K.-F., Beardsley J., Mertz D., Johnston B.C. (2017). Probiotics for the prevention of *Clostridium difficile*-associated diarrhea in adults and children. Cochrane Database Syst. Rev..

[28] Madoff S.E., Urquiaga M., Alonso C.D., Kelly C.P. (2020). Prevention of recurrent *Clostridioides difficile* infection: A systematic review of randomized controlled trials. Anaerobe.

[29] Moré M.I., Swidsinski A. (2015). *Saccharomyces boulardii* CNCM I-745 supports regeneration of the intestinal microbiota after diarrheic dysbiosis—A review. Clin. Exp. Gastroenterol..

[30] Kabbani T.A., Pallav K., Dowd S.E., Villafuerte-Galvez J., Vanga R.R., Castillo N.E., Hansen J., Dennis M., Leffler D.A., Kelly C.P. (2017). Prospective randomized controlled study on the effects of *Saccharomyces boulardii* CNCM I-745 and amoxicillin-clavulanate or the combination on the gut microbiota of healthy volunteers. Gut Microbes.

[31] Sougioultzis S., Simeonidis S., Bhaskar K.R., Chen X., Anton P.M., Keates S., Pothoulakis C., Kelly C.P. (2006). *Saccharomyces boulardii* produces a soluble anti-inflammatory factor that inhibits NF-κB-mediated IL-8 gene expression. Biochem. Biophys. Res. Commun..

[32] Stier H., Bischoff S.C. (2016). Influence of *Saccharomyces boulardii* CNCM I-745on the gut-associated immune system. Clin. Exp. Gastroenterol..

[33] Castagliuolo I., Riegler M.F., Valenick L., LaMont J.T., Pothoulakis C. (1999). *Saccharomyces boulardii* protease inhibits the effects of *Clostridium difficile* toxins A and B in human colonic mucosa. Infect. Immun..

[34] Qamar A., Aboudola S., Warny M., Michetti P., Pothoulakis C., LaMont J.T., Kelly C.P. (2001). *Saccharomyces boulardii* stimulates intestinal immunoglobulin A immune response to *Clostridium difficile* toxin A in mice. Infect. Immun..

[35] Chen X., Kokkotou E.G., Mustafa N., Bhaskar K.R., Sougioultzis S., O’Brien M., Pothoulakis C., Kelly C.P. (2006). *Saccharomyces boulardii* inhibits ERK1/2 mitogen-activated protein kinase activation both in vitro and in vivo and protects against *Clostridium difficile* toxin A-induced enteritis. J. Biol. Chem..

[36] Kelly C.P., Chong Nguyen C., Palmieri L.J., Pallav K., Dowd S.E., Humbert L., Seksik P., Bado A., Coffin B., Rainteau D. (2019). *Saccharomyces boulardii* CNCM I-745 modulates the fecal bile acids metabolism during antimicrobial therapy in healthy volunteers. Front. Microbiol..

[37] Murzyn A., Krasowska A., Stefanowicz P., Dziadkowiec D., Łukaszewicz M. (2010). Capric acid secreted by *S. boulardii* inhibits *C. albicans* filamentous growth, adhesion and biofilm formation. PLoS ONE.

[38] Vuotto C., Donelli G., Buckley A., Chilton C., Mastrantonio P., Rupnik M. (2018). *Clostridium difficile* biofilm. Updates on Clostridium Difficile in Europe: Advances in Microbiology, Infectious Diseases and Public Health Volume 8.

[39] Krasowska A., Murzyn A., Dyjankiewicz A., Łukaszewicz M., Dziadkowiec D. (2009). The antagonistic effect of *Saccharomyces boulardii* on *Candida albicans* filamentation, adhesion and biofilmf Formation. FEMS Yeast Res..

[40] Pantaléon V., Monot M., Eckert C., Hoys S., Collignon A., Janoir C., Candela T. (2018). *Clostridium difficile* forms variable biofilms on abiotic surface. Anaerobe.

[41] Carvalho F.M., Teixeira-Santos R., Mergulhão F.J.M., Gomes L.C. (2021). The use of probiotics to fight biofilms in medical devices: A systematic review and meta-analysis. Microorganisms.

[42] Karygianni L., Ren Z., Koo H., Thurnheer T. (2020). Biofilm matrixome: Extracellular components in structured microbial communities. Trends Microbiol..

[43] Flemming H.-C., Wingender J., Szewzyk U., Steinberg P., Rice S.A., Kjelleberg S. (2016). Biofilms: An emergent form of bacterial life. Nat. Rev. Microbiol..

[44] Whitchurch C.B., Tolker-Nielsen T., Ragas P.C., Mattick J.S. (2002). Extracellular DNA required for bacterial biofilm formation. Science.

[45] Okshevsky M., Meyer R.L. (2015). The role of extracellular DNA in the establishment, maintenance and perpetuation of bacterial biofilms. Crit. Rev. Microbiol..

[46] Dubois T., Tremblay Y.D.N., Hamiot A., Martin-Verstraete I., Deschamps J., Monot M., Briandet R., Dupuy B. (2019). A microbiota-generated bile salt induces biofilm formation in *Clostridium difficile*. Npj Biofilms Microbiomes.

[47] Panariello B.H.D., Klein M.I., Dias L.M., Bellini A., Costa V.B., Barbugli P.A., Pavarina A.C. (2021). *Lactobacillus casei* reduces the extracellular matrix components of fluconazole-susceptible *Candida albicans* biofilms. Biofouling.

