# Supplementary material for: Inhibition of In Vitro Clostridioides difficile Biofilm Formation by the Probiotic Yeast Saccharomyces boulardii CNCM I-745 through Modification of the Extracellular Matrix Composition
Source: Microorganisms. 2022 May 24;10(6):1082. doi: 10.3390/microorganisms10061082 (PMC9227484; doi:10.3390/microorganisms10061082)
Supplement: Supplementary file 1 [file microorganisms-10-01082-s001.zip › microorganisms-1726022-supplementary.pdf]

Supplemental data

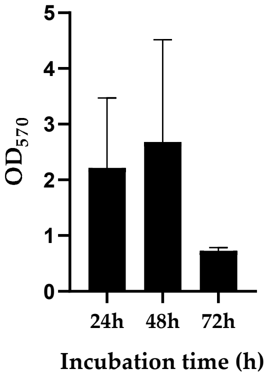

Figure S1. Biomass of mono-species biofilms produced by *S. boulardii* over time, as measured by crystal violet staining.

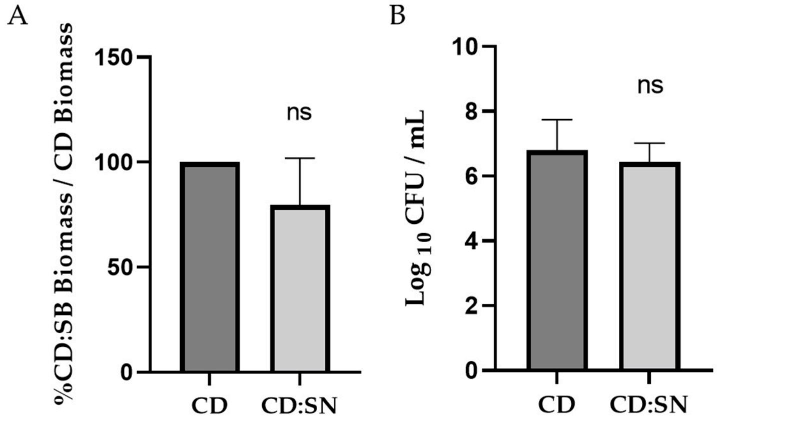

Figure S2. Impact of supernatant from *S. boulardii* culture on *C. difficile* biofilm: (A) biomass quantification; and (B) CFU counts were performed on *C. difficile* biofilm after 48 h of incubation with 500 µL of *S. boulardii* culture supernatant (SN; *S. boulardii* culture supernatant).

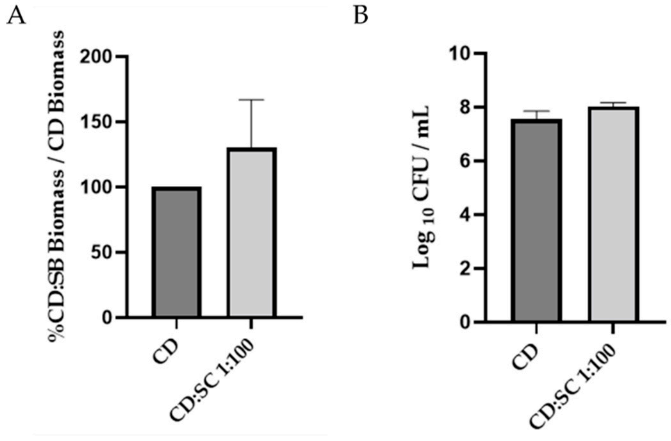

Figure S3. Biomass production (A); and bacterial VCC count (B); for 48 h dual-species biofilm formed by co-incubation of *S. cerevisiae* with *C. difficile* at a CD:Sc ratio 1:100.

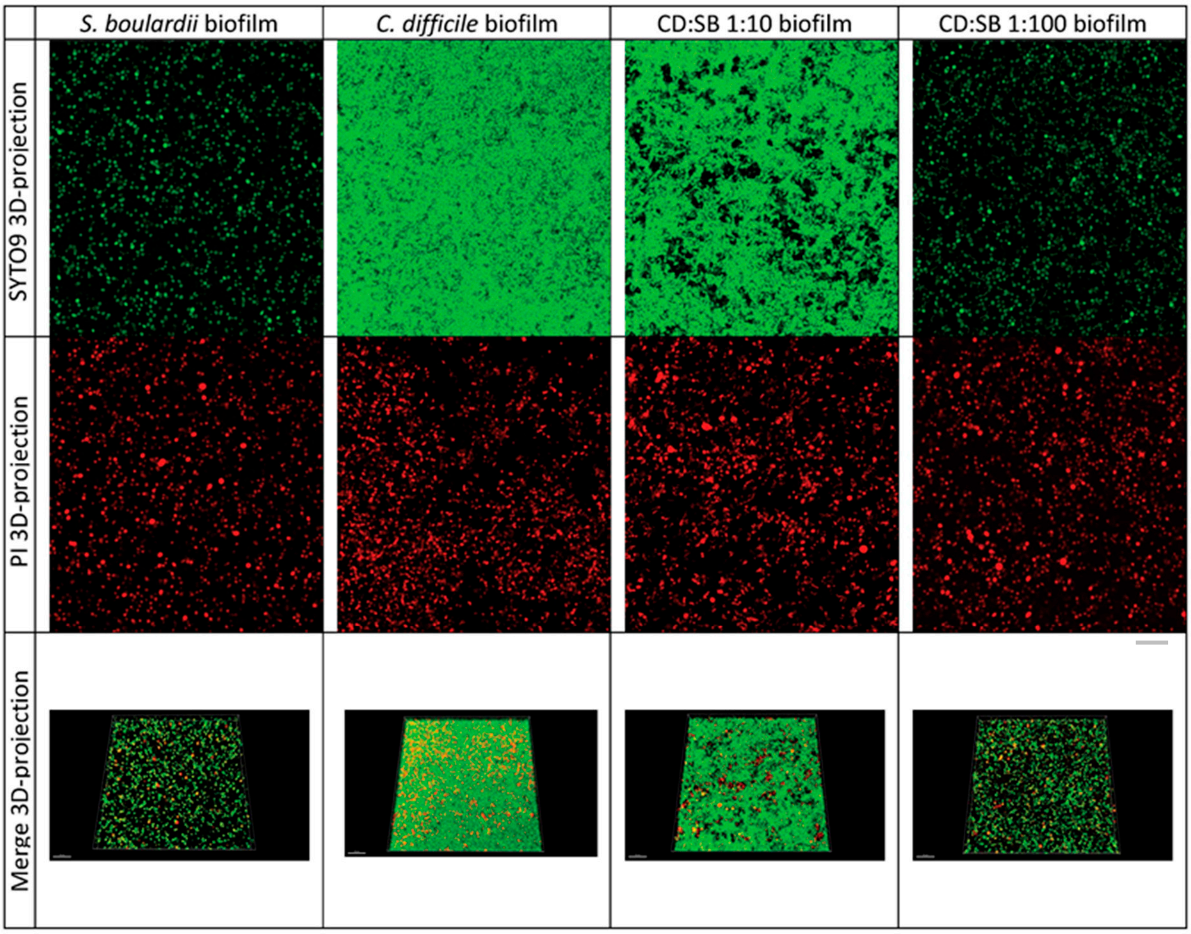

Figure S4. Representative images of CLSM observation of single-species *S. boulardii* biofilm, single-species *C. difficile* biofilm, dual-species CD + SB biofilms at CD:SB ratio 1:10 and 1:100 after 24 h of incubation; 3D representations of the biofilm stained by SYTO 9 and propidium iodide (first and second line, respectively) and a Merge representation of the 3D biofilm structures (third line) are shown (scale bar: 30 µm).

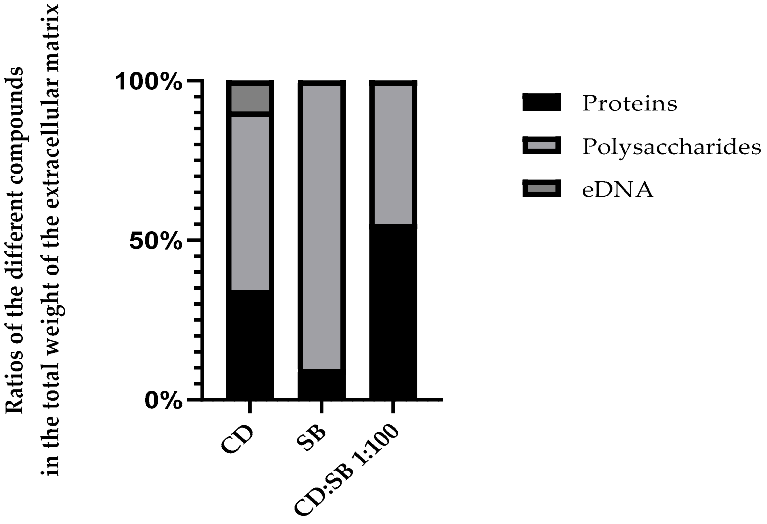

Figure S5. Ratios of polysaccharides, proteins and eDNA in the composition of extracellular matrix from single- (CD alone, SB alone) and dual-species (CD:SB, ratio 1:100) biofilms (after 48 h of incubation). The means of four independent replicates for each biofilm are shown.

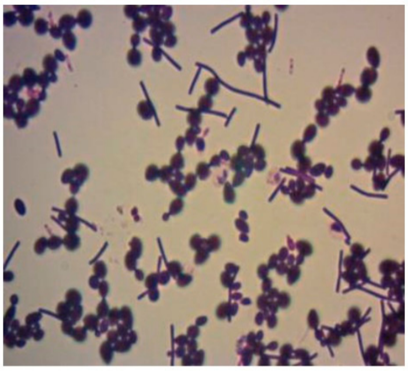

Figure S6. Optical microscopic examination after Gram staining of 24 hplanktonic co-cultures at a ratio CD:SB 1:10 (x100 magnification). This image is representative of two independent cultures. CD, strain R20291.
